# The use of mare’s milk for yogurt ice cream and synbiotic ice cream production

**DOI:** 10.1371/journal.pone.0304692

**Published:** 2024-08-07

**Authors:** Katarzyna Szkolnicka, Anna Mituniewicz-Małek, Izabela Dmytrów, Elżbieta Bogusławska-Wąs

**Affiliations:** 1 Department of Toxicology, Dairy Technology and Food Storage, Faculty of Food Sciences and Fisheries, West Pomeranian University of Technology, Szczecin, Poland; 2 Department of Applied Microbiology and Physiology of Human Nutrition, Faculty of Food Sciences and Fisheries, West Pomeranian University of Technology, Szczecin, Poland; University of Jeddah, SAUDI ARABIA

## Abstract

During the last years, growing interest in the use of mare’s milk in food production is observed. The subject of the study was to evaluate the feasibility of mare’s milk for the production of yogurt ice cream and synbiotic ice cream. Four variants of mare’s milk ice cream were developed: ice cream with yogurt bacteria without inulin (YO) and with 2% of inulin (YO+I), synbiotic ice cream with 2% inulin and *Lacticaseibacillus rhamnosus* (LCR+I) and with *Lactiplantibacillus plantarum* (LP+I). Ice creams were enriched with inulin in order to evaluate its influence on the viability of LAB and on the product quality. Physicochemical, textural and sensory analyses were performed. Count of viable bacteria cells was also evaluated. Obtained ice creams did not differ in terms of protein, fat and total solids content (1.85–1.91%, 7.33–7.58% and 24.66–26.96% respectively), but differed in acidity. Ice cream YO, the only one without inulin, had the highest acidity, what suggests that inulin decrease this parameter. Regardless the type of LAB starter culture and inulin addition, samples had the same range of overrun (35.20–44.03%) and melting rate (73.49–79.87%). However the variant of ice cream influenced textural properties and colour parameters. All obtained mare’s milk ice creams had high overall sensory quality. It was noticed, that ice cream with inulin had higher count of LAB (>7logCFU/g), than sample without inulin (>6logCFU/g). In conclusion, mare’s milk may be considered as feasible raw material for yogurt ice cream and synbiotic ice cream production.

## Introduction

Over the recent years, consumers are more and more conscious about the impact of food on health and well-being. As a result, products with high nutritional value and health promoting properties are often preferred. The global market of functional food is rising constantly and in 2020 reached over 300 billion USD. One of the biggest segments among functional foods are dairy products [1, 2]. Mare’s milk has a high potential for functional food production. Composition of mare’s milk is much more similar to human milk than cow’s milk, particularly due to low casein-to-whey protein ratio, low minerals content, high lactose content and high polyunsaturated fatty acids content [3, 4]. Furthermore, mare’s milk is a source of bioactive substances. It contains high proportion of phospholipids and PUFA n-3 fatty acids, in particular linolenic acid and it is a source of lactoferrin and lysozyme [5, 6]. Thanks to the unique composition, mare’s milk proved to have therapeutic effects. It may be useful for treatment or prevention of gastrointestinal track and respiratory system disorders [3, 7]. In addition, mare’s milk exhibits immunomodulating properties and influences intestinal microbiota composition by inhibition of pathogenic and stimulation of bifidobacteria growth [8]. This confirms, that mare’s milk is suitable for development of high quality functional food.

Currently it may be observed, that the use of mare’s milk in human nutrition increases in Europe [8, 9]. It is also the object of the studies of food technologists. Beside koumiss, a traditional fermented beverage from mare’s milk [10] is was already used for the production of such dairy products as: yogurt [5, 11, 12], kefir [13], probiotic fermented beverage [14] and ice cream [15]. To our knowledge, there is a lack of literature on the use of mare’s milk in yogurt ice cream, probiotic and synbiotic ice cream production. However it is stated, that probiotics may be successfully used for the production of functional ice cream from sheep, goat and camel milk [16–18]. Yogurt ice cream and probiotic ice cream are products combining the properties of ice cream and yogurt or probiotic milk beverage. They contain live lactic acid bacteria (LAB), which remain stable throughout the shelf life of the product [19, 20]. These types of frozen desserts may be considered as healthier ice cream alternatives.

Probiotics are strains of microorganisms that, when consumed in sufficient amounts, exhibit a positive effect on the health and well-being. Some of probiotic capabilities are cancer prevention, cholesterol reduction, and immune system modulation [21]. Microorganisms which possess probiotic characteristics mostly belong to LAB [22, 23]. Probiotics must be viable during all period of food storage and must not deteriorate its sensory and physicochemical properties. Strains of potentially probiotic *Lactiplantibacillus plantarum*, *Lacticaseibacillus casei* and other LAB like *Levilactobacillus brevis* and *Lactobacillus acidophilus* were isolated from traditional cheeses and fermented products made from raw milk [24–28]. Potentially probiotic strains of *L*. *plantarum* have high tolerance to gastrointestinal juices, bile salts and low pH and are proved to have antagonistic effects against pathogenic bacteria (*Salmonella typhimurium*, *Pseudomonas aeruginosa*, *Listeria innocula*, *Staphylococcus aureus*) and *Escherichia coli* [27, 29, 30]. Potentially probiotic strains of *L*. *casei* are also resistant to gastrointestinal conditions and have antimicrobial effect on some pathogens (*Enterobacter aerogenes*, *Listeria monocytogenes*) [28]. Some potentially probiotic strains of LAB, such as *L*. *brevis*, are able to produce gamma-aminobutyric acid (GABA). This nonprotein amino acid is a bioactive substance effective in treatment of neurological disorders [31, 32].

Probiotics are mainly introduced to fermented dairy products, such as yogurt and cheese. However, frozen dairy desserts also are products with good potential to be used as probiotic bacteria carriers [33]. In addition frozen dairy desserts are highly appreciated by consumers of all age groups. High content of milk solids and fat protects probiotic bacteria. As a result, probiotics are able to survive under freezing conditions for the long storage period [16, 17, 34].

In order to enhance functional properties of probiotic frozen dairy desserts, they may be supplemented with prebiotic, i.e. ‘substrate that is selectively used by host microorganisms and confer health benefits’ [22]. One of the prebiotics which is widely used in food industry is inulin–a kind of non-digestible dietary fibre containing naturally-occurring fructooligosaccharides [35, 36]. This oligosaccharide is fermented by microbiota in the colon and consequently assign countless natural benefits. Inulin not only stimulates positive microbiota in intestines, but also may increase the survivability of probiotic bacteria in ice cream during storage [16, 35, 37]. Beside positive impact on probiotic bacteria, inulin exhibits good technological properties when added to frozen dairy desserts. Prebiotic components improve quality parameters of ice cream, such as formation of smaller ice crystals; higher creaminess and brightness; higher overrun; lower hardness and more sweet taste [38]. In addition, inulin improves antioxidant activity of probiotic ice cream [39]. Beside fructooligosaccharides, other commonly used prebiotics are soybean oligosaccharides and galactooligosaccharides [40]. Other recently studied prebiotics are: *Spirulina platensis* microalgae extract used in probiotic yogurt sauce [41], *Arctium lappa* root used in synbiotic yogurt [40], sweet lupine powder in bio-Labneh [42] and Jerusalem artichoke tuber powder in bio-yogurt [23] and bio-frozen yogurt [20]. Moreover, in case of ice cream, the survivability of probiotic bacteria may be improved by *Stevia rebaudiana* aqueous extract addition [43]. The combination of probiotic microorganisms and prebiotics in one product leads to obtain *synbiotic* effect [16].

The study aimed to evaluate the feasibility of mare’s milk for the production of yogurt ice cream and synbiotic ice cream containing probiotic bacteria and inulin. Additionally the effect of inulin on the viability of yogurt and probiotic bacteria in mare’s milk ice creams was examined.

## Material and methods

### Materials

We purchased mare’s milk from mares milking farm located in Wielkopolskie voivodeship in Poland. Milk was obtained during summer period. Pasteurised cream from cow milk (30% of fat) and sugar were purchased at local market in Szczecin, Poland. In the study we used inulin Orafti from Beneo (Mannheim, Germany). Lactic Acid Bacteria (LAB) starter cultures YO-122, LCR and LP were obtained in freeze-dried form from Biochem s.r.l. (Roma, Italy). All used chemical reagents were analytical grade.

#### Fermented mare’s milk production

Mare’s milk was pasteurised 65°C for 30 min [12]. We used a low pasteurisation temperature to limit the inactivation of bioactive components in mare’s milk. Then milk was divided into two batches. The first one was cooled to 4±1°C and waited for ice cream production. The other one was used to prepare fermented mare’s milk. This milk was divided into 3 parts. First of them was inoculated with yogurt culture YO-122 containing *Streptococcus thermophilus* and *Lactobacillus delbrueckii* ssp. *bulgaricus* and incubated at 42°C for 4 h until pH 4.39. Second part was inoculated with LCR monoculture containing probiotic strain *Lacticaseibacillus rhamnosus* LCR and incubated at 37°C for 10 h (pH 4.35). The last one part of milk was inoculate with probiotic monoculture containing strain *Lactiplantibacillus plantarum* LP. Fermentation was carried out at 34°C for 10 h (pH 4.19). All the temperatures used for fermentation were in the range of optimum indicated by the manufacturer. The amount of freeze-dried culture added to the milk was 0.1 g/L, according to the specifications.

#### Yogurt ice cream and synbiotic ice cream production

**Fig 1:**
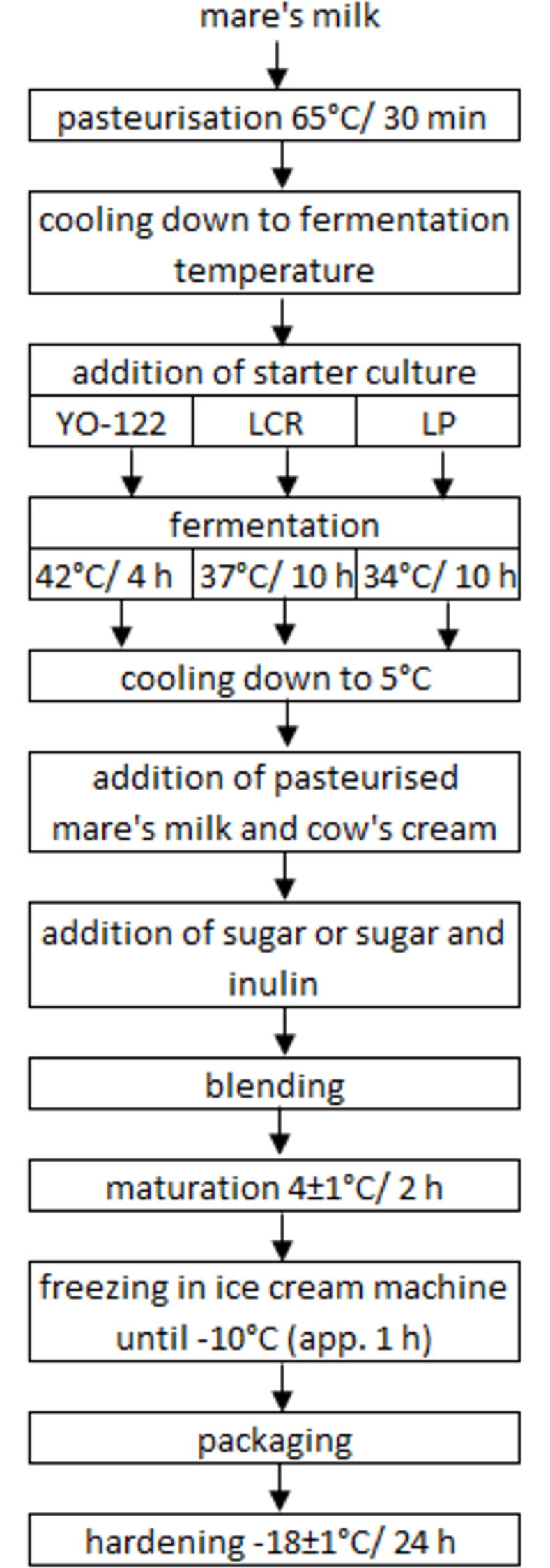
Ice cream production flowchart.

The production of ice cream was carried out in laboratory scale ice cream machine (Springlane GmbH, Germany) equipped with cooling compressor and achieving the temperature of approximately -30°C. The capacity of ice cream machine was 1.5 L. Within the study we prepared 4 kinds of ice cream: YO–with yogurt cultures; YO+I with yogurt cultures and inulin; LCR+I with *L*. *rhamnosus* LCR and inulin; LP+I with *L*. *plantarum* LP and inulin. Inulin added to ice cream replaced a part of sugar, yogurt ice cream without inulin contained 12% (w/w) of sugar and yogurt and probiotic ice cream with inulin contained 10% (w/w) of sugar and 2% (w/w) of inulin. Sensory analysis performed in preliminary studies showed, that replacing this amount of sugar with inulin does not influence considerably the sweetness of product. Moreover, according to Rezaei et al. [44], 2% inulin addition has positive impact on quality characteristics and provides appealing sensory properties of frozen yogurt. The composition of each ice cream is listed in Table 1. The production scheme is presented on Fig 1.

**Table 1:** Composition of yogurt ice creams and probiotic ice creams mixes.

Ingredients	YO	YO+I	LCR+I	LP+I
mare’s milk	33.5% (w/w)	33.5% (w/w)	33.5% (w/w)	33.5% (w/w)
fermented mare’s milk	33.5% (w/w) of yogurt with YO-122 culture	33.5% (w/w) of yogurt with YO-122 culture	33.5% (w/w) of probiotic fermented milk with LCR culture	33.5% (w/w) of probiotic fermented milk with LP culture
Cow’s cream 30% fat	21% (w/w)	21% (w/w)	21% (w/w)	21% (w/w)
sugar	12% (w/w)	10% (w/w)	10% (w/w)	10% (w/w)
inulin Orafti	-	2% (w/w)	2% (w/w)	2% (w/w)

Abbreviations: YO–yogurt ice cream; YO+I–yogurt ice cream with inulin; LCR+I–synbiotic ice cream with *Lacticaseibacillus rhamnosus* LCR and inulin; LP+I–synbiotic ice cream with *Lactiplantibacillus plantarum* LP and inulin

We used proportions presented in tab. 1 to obtain ice cream with 7% of fat, 7% of milk solids non-fat and 26% of total solids. We decided to mix fermented mare’s milk with pasteurised mare’s milk to prevent excessive acid taste of final product noticed in preliminary studies. Similar procedure combining fresh and fermented milk was carried out by Balthazar et al. [16] in the study on probiotic sheep milk ice cream. Mare’s milk used for ice cream production contained only 0.5% of fat. As the preliminary studies showed, in order to ensure proper consistency and melting resistance of frozen yogurt, cream as a source of fat was added to the mixture. Due to the fact, that mare’s milk is not used for cream production, we used cow’s cream. However mare’s milk remain the main ingredient, thus obtained product may be referred as mare’s milk ice cream. The temperature of liquid components was 5±1°C. To the liquid components, sugar and inulin were added. We did not add sugar and inulin to mare’s milk before pasteurisation to avoid the influence on fermentation process. Ice cream mixes were gently stirred with household blender, in order to minimalize shear forces, which could be a stress factor for probiotic bacteria strains [35]. Mixes were not pasteurised to retain viable bacteria. The maturation of ice cream mixes was performed at 4±1°C for 2 h. Afterwards ice cream mixes underwent the simultaneous process of freezing and aerating in ice cream machine for approximately 1 h. The temperature of ice cream directly after production reached -10°C. The samples of ice cream were packaged into unit containers suitable for freezing and stored at -18±1°C. In total, 60 samples of app. 50 g each were prepared. The analyses were carried out after 1 day from production. We assumed, that obtained ice creams may be considered as ‘craft ice cream’ made for little scale, which are intended for consumption within 1–2 days from production.

### Methods

#### Mare’s milk analyses

In mare’s milk we tested following parameters: protein (Kjeldahl method), fat (Gerber method), total solids content (drying method) and titratable acidity expressed as % of lactic acid [45]. The pH acidity was tested with a pH-meter (model HI98128, HANNA Instruments, Italy) according to the instruction manual. pH-meter was previously calibrated with buffers 4.01 and 7.01 (HANNA Instruments, Italy).

#### Ice cream analyses

The determination of pH (pH-meter HI98128, HANNA Instruments, Italy), titratable acidity presented as % lactic acid, fat, protein and total solids content were performed after tawing of ice cream samples in water bath at 25°C. The methods of analyses were in accordance with AOAC [45] and were similar to those performed for milk.

Overrun of ice cream is a parameter informing us how much the volume of ice cream mix increases during the freezing. It is connected with the volume of incorporated air. Overrun was calculated as a difference between the weight of equal volume of ice cream mix and ice cream in accordance to the following formula [35]:

$$\text{Overrun}[\%]=\frac{\text{weight}\;\text{of}\;\text{ice}\;\text{cream}\;\text{mix-weight}\;\text{of}\;\text{ice}\;\text{cream}}{\text{weight}\;\text{of}\;\text{ice}\;\text{cream}}\times 100$$ (1)

Meltdown rate [%] was determined according to the method presented by Milani and Koocheki [46] with some modifications [47]. A scoop of ice cream with the weight of app. 25 g was put on a 1 mm plastic mesh placed on a beaker. The samples were incubated at 20±0.1°C for 60 min. During this time sample was melting and dropping to the beaker, which then was weighed. Meltdown rate was calculated according to the following formula and was expressed as percent of the initial weight of ice cream scoop:

$$\text{Meltdown}\;\text{rate}[\%]=\frac{\text{weight}\;\text{of}\;\text{melted}\;\text{sample}}{\text{weight}\;\text{of}\;\text{scoop}}\times 100$$ (2)

The texture profile analysis (TPA) was carried out with the use of texture analyser TA.XT plus (Stable Micro System, Great Britain). Within the test, two parameters were measured: hardness, which is defined as the peak force during the sample penetration and adhesiveness, which is defined as the negative peak force during the probe withdrawal. Cylindrical aluminium probe with 10 mm diameter was used. The trigger force was 5 g, penetration distance was 25 mm and test speed was 5 m/s. Before analysis was performed, the equipment was calibrated in terms of force and height. To simulate the conditions under which ice creams are consumed, the measurement was conducted at ambient temperature (20±1°C) and the samples were previously tempered at -6±1°C for 1 h [47].

Colour was tested on the surface of ice cream samples using the CIELAB system. The colour parameters were: L* (lightness), a* (−green/+red colour), and b* (−blue/+yellow colour) [4]. We used colorimeter (model FRU® WR-18, Shenzhen Wave Optoelectronics Technology Co., Ltd, China) with 8 mm aperture. Before the test, the colorimeter was calibrated with the use of standard white plate.

Sensory evaluation was performed by a panel of 6 evaluators (4 female and 2 male), workers of Faculty of Food Sciences and Fisheries of West Pomeranian University of Technology in Szczecin, Poland. The assessors were trained in dairy product assessment [48, 49]. Within the evaluation, the overall sensory quality and sensory profile analysis with QDA method were assessed. The overall sensory quality was analysed by grading the sensory characteristics: consistency; taste; flavour; appearance and colour in the range from 1 (very poor) to 5 points (very good quality). By summing up the points, we calculated overall sensory quality which maximally could reach 20 points. In quantitative descriptive analysis (QDA) method, the panellists had to rate the sensory attributes of ice cream using 9-point scale with 0 –lack of attribute, 1 –the lowest intensity and 9 –the highest intensity. The sensory attributes were: colour, colour uniformity, uniform consistency, creaminess, hard structure, smoothness, presence of clumps/granules, cream aroma, acidity, bitterness and foreign aroma (Table 2). The attributes were chosen by evaluators during training session. The samples were coded with random three-digit numbers. The temperature of samples during serving was -10±2°C. The analysis was performed in sensory analysis laboratory in which each assessors had separate stand and received water to clean the palate [47, 50–52].

**Table 2:** Attributes of ice cream sensory analyses with QDA method [47].

Attribute	Definition	Scale
*assessment of overall sensory quality (1–5)*		
consistency	the liking of consistency	1 ‐ extremely dislike, 5 ‐ extremely like
taste	the liking of taste	1 ‐ extremely dislike, 5 ‐ extremely like
flavour	the liking of flavour	1 ‐ extremely dislike, 5 ‐ extremely like
colour	the liking of colour	1 ‐ extremely dislike, 5 ‐ extremely like
appearance	the liking of appearance	1 ‐ extremely dislike, 5 ‐ extremely like
*sensory profile analysis (0–9)*		
colour	white to dark ivory colour	0 ‐ white, 9 ‐ dark ivory
colour uniformity	uniform colour in all sample	0 ‐ totally ununiform, 9 ‐ totally uniform
uniform consistency	uniform and homogenous consistency	0 ‐ totally ununiform, 9 ‐ totally uniform
creaminess	creamy, easy to scoop consistency	0 ‐ lack of attribute, 9 ‐ intensive attribute
hard structure	hard, difficult to scoop structure	0 –very soft structure, 9 –very hard structure
smoothness	smooth and even consistency	0 ‐ lack of attribute, 9 ‐ intensive attribute
presence of clumps/granules	presence of particles detectable in mouth	0 ‐ lack of attribute, 9 ‐ intensive attribute
cream aroma	aroma associated with creamy products	0 ‐ lack of attribute, 9 ‐ intensive attribute
acidity	taste sensation typical for products with lactic acid	0 ‐ lack of attribute, 9 ‐ intensive attribute
bitterness	sharp taste typical for caffeine or quinine	0 ‐ lack of attribute, 9 ‐ intensive attribute
foreign aroma	presence of off-flavours typically not present in ice cream	0 ‐ lack of attribute, 9 ‐ intensive attribute

#### Microbiological analysis

Microbiological analysis was performed for ice creams and fermented mare’s milk used for their production. To prepare the samples, we followed the procedure in accordance with Standard [53]. Lactic acid bacteria: *Streptococcus thermophilus*, *Lactobacillus delbrueckii* subsp. *bulgaricus*, *Lacticaseibacillus rhamnosus* and *Lactiplantibacillus plantarum* were determined according to Standard [54]. In the study following media for lactic acid bacteria were used: M-17 (BTL, Poland) for cocci and MRS agar (Oxoid, UK) for bacilli, respectively. Incubation was carried out at a temperature suitable for the determined microorganisms, according to the data provided by bacterial cultures producer (Biochem s.r.l., Roma, Italy). The count of the microorganisms was expressed as log cfu/g.

#### Statistical analysis

Results were statistically analysed using Statistica 13.1 Software (StatSoft Inc., USA) at the significance level p < 0.05. Mean values and standard deviations were calculated. All factors had normal distribution of variables, what was stated with the use of Shapiro-Wilk’s test. The differences among the samples were analysed by one-way ANOVA and Tukey’s post hoc test. Depending on the methodology, analyses were performer in 3–6 repetitions.

## Results and discussion

Mare’s milk composition was as follow: fat content 0.50±0.00%; protein content 2.37±0.06%; total solids 8.24±0.18%. Titratable acidity expressed as percentage of lactic acid was 0.142±0.003% and pH was 6.70±0.05. The density of mare’s milk was 1.030±0.001 g.cm^-1^. According to the literature, mare’s milk composition may vary in wide ranges. Claeys et al. [55] claims that fat and protein content are within the ranges 0.3–4.2% and 1.4–3.2% respectively. Potočnik et al. [4] gives different ranges: 0.5–2.0% for fat and 1.5–2.8% for protein. Our results are within both of these this ranges. Teichert et. al [5] found similar to our protein content (2.29%) and higher fat and total solids content (1.32% and 9.73% respectively). The same authors stated higher than in our study pH value of 6.92. Cagno et al. [56] found much higher than in our study fat content of 1.93% and lower protein content of 2.05%, they also found higher pH 7.0. Very close to our results protein content 2.39%, was demonstrated by Cais Sokolińska et al. [13]. According to Dmytrów and Włodarczyk [57], the chemical composition and physicochemical properties of mare’s milk are influenced by genetic (species, breed) and non-genetic factors, i.e. the age of the animal, previous pregnancies and lactations, climatic conditions, farming conditions and feeding.

Table 3 presents pH, titratable acidity and chemical composition of ice creams from mare’s milk. Significantly the lowest pH and the highest lactic acid content had variant YO. The sample with the lowest acidity was LCR+I. Ice creams YO+I and LP+I did not differ in acidity. The type of bacteria culture as well as the inulin addition did not influence significantly the protein, fat and total solids content. In all samples, the content of protein was slightly below 2%, fat content exceeded 7%, and the level of total solids was up to 26.96%. Low fat content is an advantage because of lower calorie content in ice cream. According to Mostafavi [51], ice cream with about 7.5% of fat may be classified as reduced fat. In the study conducted by Akanova et al. [15], the content of protein and fat in mare’s milk ice cream was respectively 2.5 and 2.1%. Because the literature on mare’s milk ice cream is very limited, it is reasonable to compare our results with the results describing ice cream made from other types of milk. Balthazar et al. [16] analysed probiotic ice cream with *L*. *casei* and inulin made from sheep’s milk. The pH value of sample without inulin was 5.30 and with 10% of inulin was 5.70. The increase of pH caused by inulin addition is in line with our findings (Table 3). However, in the study of Falah et al [39] on probiotic ice cream with *Levilactobacillus brevis* and inulin in concentration of 0, 1.5 and 3%, prebiotic addition did not remarkably changed the acidity. Akalin & Erisir [58] tested probiotic ice cream from cow’s milk with 4% of inulin, 4% of fat and 33.5% of total solids. The pH acidity of the product was 5.35, what is slightly lower than in our study and lactic acid content was 0.52%. Probiotic frozen yogurt from cow’s milk with similar fat and total solids content (8% and 33.25% respectively) was the subject of the study of Atallah et al. [19] and the content of lactic acid was 0.44%.

**Table 3:** Titratable acidity, pH, protein, fat and total solids in yogurt and synbiotic mare’s milk ice creams.

Characteristic	Kind of mare’s milk ice cream				one-factor ANOVA	
	YO	YO+I	LCR+I	LP+I	F-value	p-value
**pH**	5,47 ^a^ ±0,02	5,53 ^b^ ±0,03	5,65 ^c^ ±0,01	5,52 ^b^ ±0,01	69,60000	0,000004
**% of lactic acid**	0,28 ^c^ ±0,01	0,25 ^b^ ±0,01	0,20 ^a^ ±0,00	0,23 ^b^ ±0,00	72,44444	0,000004
**Protein, %**	1,91 ^a^ ±0,04	1,86 ^a^ ±0,09	1,85 ^a^ ±0,03	1,89 ^a^ ±0,15	0,24986	0,859299
**Fat, %**	7,58 ^a^ ±0,14	7,33 ^a^ ±0,14	7,35 ^a^ ±0,13	7,33 ^a^ ±0,14	2,25000	0,159767
**Total solids, %**	24,66 ^a^ ±2,63	26,96 ^a^ ±1,44	25,99 ^a^ ±3,07	25,58 ^a^ ±1,34	0,53788	0,66943

Table presents mean value ± standard deviation.

Abbreviations: YO–yogurt ice cream; YO+I–yogurt ice cream with inulin; LCR+I–synbiotic ice cream with *Lacticaseibacillus rhamnosus* LCR and inulin; LP+I–synbiotic ice cream with *Lactiplantibacillus plantarum* LP and inulin

Different letters in superscript indicate statistically significant (p < 0.05) differences between mean values in rows.

Effect of different bacteria cultures and inulin addition on the value of overrun, melting rate and texture parameters of mare’s milk ice cream is shown in Table 4. Many authors confirm, that inulin is desirable component of ice creams based on yogurt, namely it works as a fat replacer; increases the viscosity of ice cream mixes; improves melting resistance, increases overrun and hardness of ice cream [35, 58, 59]. However in present study, all obtained samples did not differ significantly in the value of overrun and melting rate. High values of melting rate observed in our study may be connected with low fat content, because this ice cream component positively affects melting resistance of product [59]. Overrun values obtained by other authors are differentiated. Akin et al. [35] who tested probiotic ice cream with 16% sugar and 2% inulin obtained 36.5% of overrun what is in the same range that in our study. Rezaei et al. [44], who examined probiotic ice cream with 16% of sugar and 0, 1 or 2% of inulin revealed, that inulin addition influenced overrun value. Sample without inulin had 22.3% overrun and with 2% of inulin 38.5%. The same authors stated, that inulin addition did not influence melting rate, which was in range 84.5–96.4%, what is higher than in our study. In turn the values of overrun and melting rate of low-fat probiotic ice cream with 4% inulin studied by Akalin & Erisir [58] was 50.6% and about 50% respectively. Higher values of overrun than in our study were stated also by Atallah et al. [19] in cow’s milk probiotic frozen yogurt (57.1% - 59.9%) and by Balthazar et al. [16] in sheep’s milk ice cream (77.5% - 87%). Overrun is one of the crucial characteristics of ice creams providing their light texture and influencing melting properties [34, 44, 60]. However low overrun is positive for bacteria. Excessive oxygen caused by air incorporation negatively affects growth of microaerophilic lactic acid bacteria [35]. Overrun and melting behaviour of ice cream is determined by the mix composition and production conditions. Moreover, these properties are positively affected by the presence of stabilizers, which we did not use in present studies.

**Table 4:** Overrun, melting rate and texture parameters of yogurt and synbiotic mare’s milk ice creams.

Characteristic	Kind of mare’s milk ice cream				one-factor ANOVA	
	YO	YO+I	LCR+I	LP+I	F-value	p-value
**Overrun, %**	43,62 ^a^ ±5,88	44,03 ^a^ ±5,97	37,49 ^a^ ±7,22	35,20 ^a^ ±9,33	1,49648	0,265467
**Melting rate, %**	73,49 ^a^ ±2,67	76,97 ^a^ ±1,00	79,87 ^a^ ±2,59	74,30 ^a^ ±3,43	3,77692	0,058969
**Hardness, N**	0.34 ^a^ ±0.03	0.37 ^a^ ±0.04	0.79 ^b^ ±0.09	1.14 ^c^ ±0.12	125.5448	0.000000
**Adhesiveness, g.sec**	91.19 ^a^ ±20.04	83.54 ^a^ ±8.10	160.03 ^b^ ±22.65	165.64 ^b^ ±21.17	27.3131	0.000002

Table presents mean value ± standard deviation.

Abbreviations: YO–yogurt ice cream; YO+I–yogurt ice cream with inulin; LCR+I–synbiotic ice cream with *Lacticaseibacillus rhamnosus* LCR and inulin; LP+I–synbiotic ice cream with *Lactiplantibacillus plantarum* LP and inulin

Different letters in superscript indicate statistically significant (p < 0.05) differences between mean values in rows.

The values of hardness and adhesiveness, pivotal textural attributes of ice cream, varied between samples (Table 4). Probiotic mare’s milk ice cream were characterised with higher hardness and adhesiveness than yogurt ice creams. Similar range of hardness (0.3–0.7 N) in lactose-free cow’s milk frozen yogurt was stated by Skryplonek et al. [61]. However different authors stated much higher values of hardness of ice cream. Atallah et al. [19] stated hardness in the range 50.32–52.60 N in probiotic cow’s milk ice cream and Balthazar et al. [16] in the range 42.58–88.01 N in probiotic sheep’s milk ice cream. This discrepancy is most probably connected with different raw material as well as differences in methodology of the texture analysis. Similarly like in case of overrun and melting properties, the texture of ice cream is connected with both mix composition and production conditions. The decrease of fat and sugar content results in higher hardness and lower adhesiveness of final product [61].

**Fig 2:**
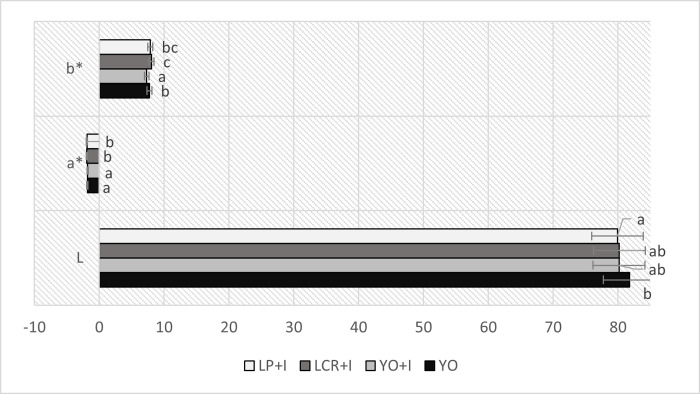
Colour parameters (L*, a*, b*) of yogurt and synbiotic mare’s milk ice creams. Abbreviations: YO–yogurt ice cream; YO+I–yogurt ice cream with inulin; LCR+I–synbiotic ice cream with *Lacticaseibacillus rhamnosus* LCR and inulin; LP+I–synbiotic ice cream with *Lactiplantibacillus plantarum* LP and inulin Different letters indicate statistically significant (p < 0.05) differences.

Fig 2 presents colour parameters of obtained mare’s milk ice creams. Colour of food products is one of crucial factors determining consumer’s acceptance [62]. The type of mare’s milk ice cream influenced its colour. However the colour of all samples was natural and attractive. It was creamy white, with greenish-yellowish hue, as evidenced by negative values of parameter a* (green) and positive values of parameter b* (yellow). The most bright (the highest L value) was ice cream YO without inulin addition. Ice cream LCR+I was characterized with the lowest a* value (-1.93) and the highest b* value (8.07). According to Cais-Sokolińska and Pikul [62], who analysed the colour of yogurt, the lightness of the product is affected by the acidity and the increase of acidity causes the decrease of lightness. Analogical observation in relation to mare’s milk can be found in work of Teichert et al. [5]. This statement is not in line with our findings, because sample YO with the highest acidity had also the highest lightness index. The lightness of lactose-free frozen yogurt from cow’s milk obtained by Skryplonek et al. [61] was higher than our results and exceeded 90. The L index of probiotic sheep ice cream studied by Balthazar et al. [16] ranged 93.00–94.84 and was also distinctly higher than in case of mare’s milk ice cream.

**Fig 3:**
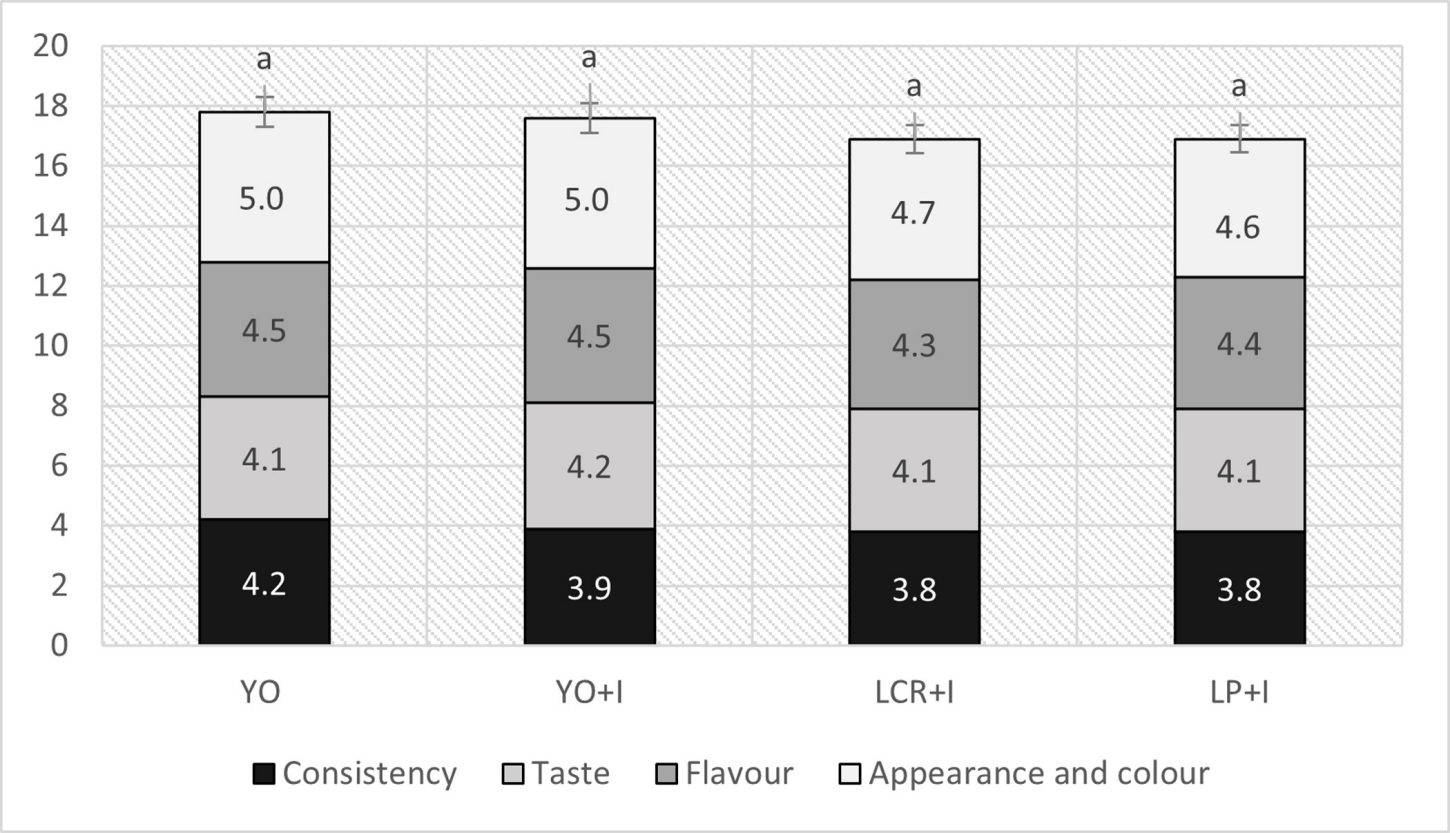
Overall sensory quality of yogurt and synbiotic mare’s milk ice creams. Abbreviations: YO–yogurt ice cream; YO+I–yogurt ice cream with inulin; LCR+I–synbiotic ice cream with *Lacticaseibacillus rhamnosus* LCR and inulin; LP+I–synbiotic ice cream with *Lactiplantibacillus plantarum* LP and inulin Different letters indicate statistically significant (p < 0.05) differences.

While introducing new food product to the market, the sensory attractiveness is crucial aspect influencing the product success [63]. Fig 3 presents the overall sensory quality of mare’s milk ice cream and the maximal value is 20 points. The bars representing overall sensory quality of ice cream are divided into segments representing the values of consistency, taste, flavour, appearance and colour. Overall sensory quality of the samples did not differ significantly (p > 0.05) (Table 5) and ranged 16.90–17.80 points, what may be perceived as hight score.

**Table 5:** Results of one-factor analysis of variance.

Characteristic	one-factor ANOVA	
	F-value	p-value
*Colour parameters*		
L	3,9073*	0,023951*
a*	24,1471*	0,000001*
b*	19,8474*	0,000003*
*Sensory properties*		
overall sensory quality	0,3235	0,808306
colour	0,3158	0,813764
colour uniformity	x	x
uniform consistency	0,2303	0,873924
creaminess	0,3664	0,778180
hard structure	0,0670	0,976634
smoothness	0,0460	0,986427
presence of clumps/granules	0,1366	0,936270
cream aroma	0,0509	0,984275
acidity	6,4478*	0,004549*
bitterness	x	x
foreign aroma	x	x
*Count of lactic acid bacteria*		
in fermented milk	14.653*	0.000028*
in ice cream	1195.384*	0.000000*

Explanatory note:

* ‐ type of sample significantly influenced tested characteristic; x ‐ lack of the result due to standard deviation = 0.

**Fig 4:**
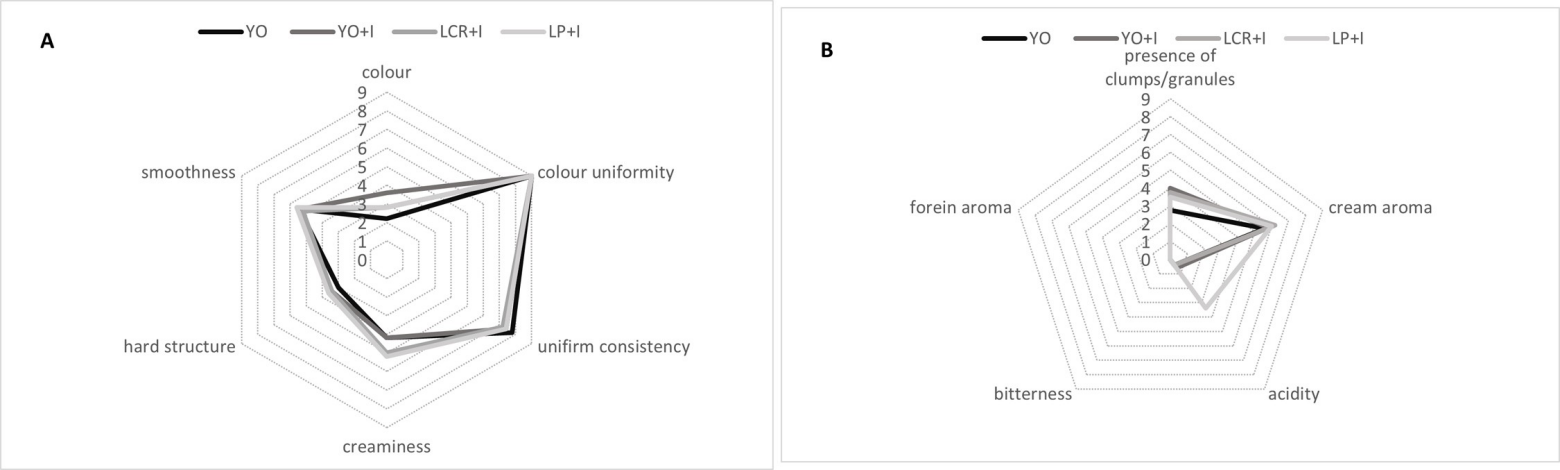
A and B. Sensory profile analysis with QDA method of yogurt and synbiotic mare’s milk ice creams. Abbreviations: YO–yogurt ice cream; YO+I–yogurt ice cream with inulin; LCR+I–synbiotic ice cream with *Lacticaseibacillus rhamnosus* LCR and inulin; LP+I–synbiotic ice cream with *Lactiplantibacillus plantarum* LP and inulin.

The results of quantitative descriptive analysis (QDA) are showed on Fig 4, where A presents the attributes connected with colour and consistency, and B shows the presence of clumps/ granules, the taste and flavour of mare’s milk ice creams. The colour of all samples was perceived as white-creamy and uniform. The texture was assessed as soft, quite creamy and slightly coarse. All samples had pleasant, creamy taste. Statistical analysis revealed, that the only attribute in term of which the samples differed significantly was acidity (p < 0.05) (Table 5) and ice cream LP+I with inulin and *L*. *plantarum* had distinctly more acid flavour than other samples. The presence of slightly coarse consistency in obtained ice creams may be connected with lower fat content than in full fat products, which contains about 10% fat. According to Mostafavi [51] reduction of fat in ice cream formulation results in less creamy consistency and lower mouth-coating properties, what enhance the perception of ice crystals in the product. Good sensory quality of obtained mare’s milk ice cream may be connected with the presence of inulin, which may improve the total acceptance of frozen dairy dessert [36]. In the study of Rezaei et al. [44] on cow’s milk probiotic frozen yogurt with 0%, 1% or 2% inulin, the highest inulin addition (2%) resulted in the most appealing sensory characteristics. Improved sensory properties of probiotic ice cream with 3% inulin was noticed also in the study of Falah et al. [39]. On the other hand, the sensory properties of samples YO and YO+I without and with 2% inulin addition respectively, did not differ. It is in line with findings of Akin et al. [35] who stated, that 1% and 2% inulin addition to probiotic ice cream has no effect on the sensory properties. In study of Falah et al. [21] 2.5% addition of inulin to synbiotic cow’s milk yogurt increased sensory evaluation score by improvement of textue and flavour.

**Fig 5:**
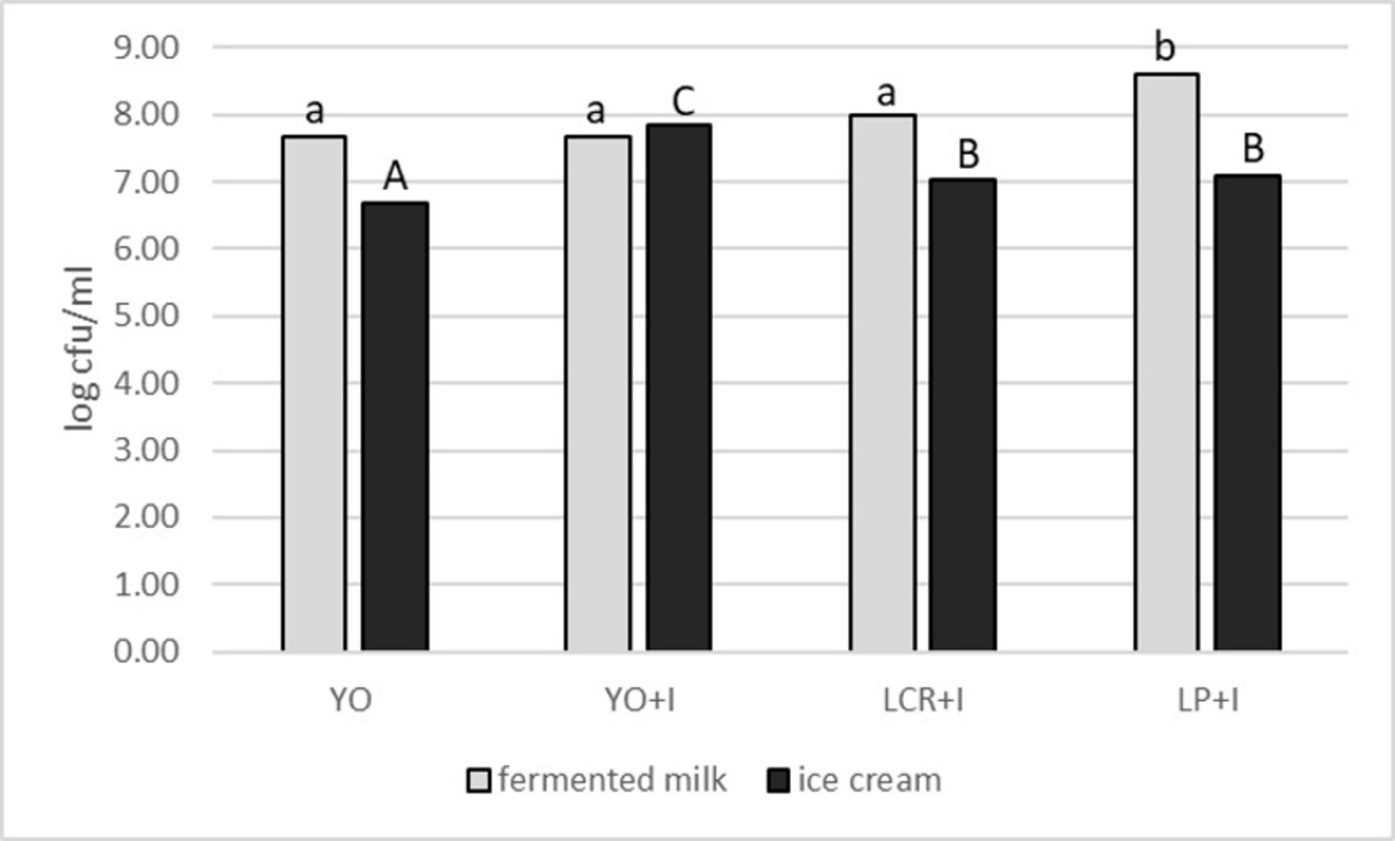
Count of lactic acid bacteria in yogurt and synbiotic mare’s milk ice creams and in fermented mare’s milk used for their production. Abbreviations: YO–yogurt ice cream; YO+I–yogurt ice cream with inulin; LCR+I–synbiotic ice cream with *Lacticaseibacillus rhamnosus* LCR and inulin; LP+I–synbiotic ice cream with *Lactiplantibacillus plantarum* LP and inulin. Different letters indicate statistically significant (p < 0.05) differences within fermented milk (a,b) or ice cream (A,B,C).

Important parameter influencing the quality of fermented and especially probiotic dairy products is the count of viable cells of bacteria [17]. In present studies all variants were produced with the use of lactic acid bacteria. Two variants of ice cream contained strains of yogurt bacteria *S*. *thermophilus* and *L*. *delbrueckii* subsp. *bulgaricus* and another two, strains of probiotic lactic acid bacteria *L*. *rhamnosus* and *L*. *plantarum*. The counts of LAB in fermented mare’s milk used for mix preparation and in ice cream are presented in Fig 5. Inulin addition improved the survivability of yogurt bacteria during process of ice cream production. Initial value of yogurt bacteria strains in fermented mare’s milk was 7.67 log cfu/ml. Similar count of yogurt bacteria in mare’s milk fermented beverage, exceeding 7 log cfu/ml was found by Teichert et al. [5]. The count of yogurt bacteria obtained by Cagno et al. [56] in fermented mare’s milk was about 6 log cfu/ml of *L*. *delbrueckii* subsp. *bulgaricus* and 8 log cfu/ml of *S*. *thermophilus*. In ice cream YO the number of bacteria reducted to 6.69 log cfu/ml In case of ice cream YO+I with inulin addition, the bacteria count was in the same range that in fermented milk before ice cream production. It indicates the property of inulin to protect LAB during the process of freezing. Smaller drop of LAB count in frozen yogurt containing 2% inulin in comparison to control frozen yogurt was noted also by Rezaei et al. [44]. In case of synbiotic samples, mare’s milk fermented by *L*. *rhamnosus* (LCR+I) and *L*. *plantarum* (LP+I) were characterised by high bacteria content (8.00 and 8.59 log cfu/ml respectively). This results proved, that mare’s milk is good environment for probiotics. It may be connected with high lactose content, which is a substrate for probiotic bacteria. The process of ice cream production resulted in a reduction of the count of probiotic bacteria for about 1 log to the value 7.02 log cfu/ml for LCR+I and 7.08 log cfu/ml for LP+I. Observed decline of LAB count may be caused by combination of fermented mare’s milk with pasteurised milk and other ice cream mix components, what diluted the bacteria. Significant reduction in *L*. *acidophilus* LA-5 during freezing of ice cream mix prepared from camel’s milk was observed by Elkot et al. [18]. The values of probiotic bacteria count are distinctly higher than required minimal therapeutic level which amounts 6.0 log cfu/ml [64]. Balthazar et al. [16] in the study on probiotic sheep ice cream with *L*. *casei* and 10% inulin also obtained sufficient count of probiotics, which was 7.90 log cfu/ml in mix and 7.86 log cfu/ml in ice cream.

High survivability of LAB during the production of ice cream proves, that this product is good carrier of lactic acid bacteria. It is due to the presence of fat, casein, sucrose and lactose, which play the role of cryoprotective agents [17, 44]. Observed reduction in the count of LAB during ice cream production is connected with the process of air incorporation and freezing [17]. The overrun is considered as destructive factor especially for probiotic bacteria which are mostly microaerophilic [34, 44]. Cold shock and osmotic pressure occurring during freezing may also impair bacteria. Furthermore mechanical stress during ice cream mix homogenisation and ice crystals formation are another stress factors [44]. Among the samples of mare’s milk ice creams, the lowest amount of LAB was found in sample YO, the only one without 2% inulin addition. This results are in line with studies of other authors [16, 21, 39, 44, 59], who proved positive impact of inulin on the viability of lactic acid bacteria in frozen dairy desserts.

## Conclusions

Mare’s milk is suitable raw material for yogurt ice cream and synbiotic ice cream production, which due to unique composition of mare’s milk may be perceived as valuable functional food, bringing benefits to consumer’s health. Mare’s milk ice cream are good carrier for yogurt and probiotic bacteria *L*. *rhamnosus* LCR and *L*. *plantarum* LP. The count of probiotics after the production process exceeds the required therapeutical level (6.0 log cfu/g). Count of viable LAB in samples with inulin addition was above 7.0 log cfu/g. In sample without inulin the count of bacteria was significantly lower (above 6.0 log cfu/g), what indicate positive impact of inulin on LAB survivability in mare’s milk ice cream. Type of ice cream influenced pH and titratable acidity. All variants of mare’s milk ice cream had low hardness (0.34–1.14 N) and high overrun (35.20–44.03%) what is linked with good sensory attractiveness [39]. High value of melting rate may be connected with low fat content in ice cream. Inulin modified the colour parameters of mare’s milk frozen desserts. Ice creams did not differ in term of sensory characteristics and all have high overall sensory quality.

Obtained products seem to be a good candidates to introduce mare’s milk to the diet of western consumers, which are not familiar with this milk type. New mare’s milk products can contribute to the increment of the production and processing of this milk type.

## Supporting information

S1 Data (XLSX)
